# Long-term metabolic risks after thyroidectomy and thyroid hormone therapy in thyroid cancer survivors: a nationwide cohort study

**DOI:** 10.3389/fendo.2026.1845515

**Published:** 2026-09-08

**Authors:** Jiwoo Lee, Chung Ho Kim, Yeonjae Kim, Yeonjung Ha, Yun Mi Choi, Bomi Park

**Affiliations:** 1 Department of Internal Medicine, Hallym University Dongtan Sacred Heart Hospital, Hallym University College of Medicine, Hwaseong, Republic of Korea; 2 Department of Preventive Medicine, College of Medicine, Chung-Ang University, Seoul, Republic of Korea; 3 Department of Internal Medicine, CHA Bundang Medical Center, CHA University, Gyeonggi-do, Republic of Korea

**Keywords:** metabolic disorders, thyroid cancer (TC), thyroid cancer survivors, thyroid hormone therapy, thyroidectomy

## Abstract

**Background:**

As long-term survival rates improve among thyroid cancer patients, the metabolic consequences of thyroidectomy and thyroid hormone therapy have gained increasing importance. While these treatments are crucial for cancer management, their long-term effects on metabolic health remain inadequately understood.

**Methods:**

We conducted a retrospective, population-based cohort study utilizing nationwide data from the Korea National Health Insurance Service. We estimated the risk of incident metabolic abnormalities (i) among patients with TC who underwent thyroidectomy compared with matched individuals from the general population without TC, (ii) among TC patients according to thyroidectomy status, and (iii) among TC patients who underwent thyroidectomy, according to thyroid hormone therapy status. The incidence of obesity, diabetes mellitus, hypertension, dyslipidemia, metabolic syndrome, and metabolic dysfunction-associated steatotic liver disease (MASLD) within 5, 8, and 10 years was estimated using multivariable logistic regression models, adjusting for age, sex, household income, alcohol consumption, smoking status, and physical activity.

**Results:**

Thyroidectomy was associated with higher odds of multiple metabolic abnormalities compared with the matched general population without TC and to TC patients without thyroidectomy, with most associations evident from 5 years onward and some from 8 years. Among TC patients who underwent thyroidectomy, thyroid hormone therapy was significantly associated with metabolic abnormalities. In sex-stratified analyses, overall patterns were largely similar; however, MASLD was not significantly associated with thyroidectomy or thyroid hormone therapy in males.

**Conclusion:**

Thyroidectomy and thyroid hormone therapy are associated with incidence of metabolic abnormalities within 5, 8, or 10 years among thyroid cancer survivors. These findings support ongoing metabolic monitoring in thyroid cancer survivors and warrant further studies to determine whether individualized hormone management can reduce long-term metabolic risk.

## Introduction

Thyroid cancer (TC) is the most prevalent endocrine malignancy globally (1), with South Korea exhibiting the highest incidence, largely attributed to extensive ultrasound screening (2). Although differentiated TC generally presents an excellent prognosis and exhibits low disease-specific mortality, the increasing number of survivors has shifted clinical focus towards the long-term health implications (3, 4). Surgical resection, whether through total thyroidectomy or lobectomy, remains the primary treatment, with thyroid hormone replacement frequently administered to suppress thyroid-stimulating hormone (TSH) levels (5). While these therapeutic approaches are effective in an oncological context, their broader systemic effects—particularly concerning metabolic health—remain inadequately understood (6).

Thyroid hormones regulate metabolic rate, glucose and lipid metabolism, and cardiovascular function (7, 8). Disruption of thyroid homeostasis, resulting from surgical removal of the thyroid or pharmacological replacement therapy, may predispose patients to metabolic dysfunction (9). Previous studies have indicated associations between altered thyroid function and conditions such as obesity, insulin resistance, dyslipidemia, hypertension, and metabolic dysfunction-associated steatotic liver disease (MASLD) (10–14). Even subclinical hypothyroidism has been linked to dyslipidemia and impaired glucose tolerance (15, 16), while hyperthyroidism has been associated with muscle wasting and arrhythmias (17, 18).

Despite these findings, the specific long-term metabolic consequences of thyroidectomy and chronic thyroid hormone therapy remain poorly characterized. Current evidence is limited by small sample sizes and short follow-up durations (19, 20), and few studies have distinguished the independent contributions of surgery, underlying TC status, and prolonged hormone therapy on metabolic risk (21–23).

To address these gaps, we conducted a nationwide retrospective cohort study using the Korea National Health Insurance Service (KNHIS) database, which integrates claims data with standardized health screening information. We examined the incidence of obesity, diabetes mellitus, hypertension, dyslipidemia, metabolic syndrome, and MASLD over 5-, 8-, and 10-year follow-up periods, using three complementary comparisons: (1) patients with TC who underwent thyroidectomy compared with matched individuals from the general population without TC, (2) patients with TC according to thyroidectomy status, and (3) patients who underwent thyroidectomy according to thyroid hormone therapy status.

## Materials and methods

### Study population

The present study was conducted as a retrospective, population-based cohort study utilizing customized nationwide data from the KNHIS. The KNHIS database includes eligibility information for nearly the entire Korean population and comprehensive healthcare utilization data from medical institutions and pharmacies nationwide (24). The customized KNHIS data utilized in this study included information on healthcare utilization, disease diagnosis codes, treatment and procedure codes, and prescription drug records. These claims data were linked to records from the Korean National Health Screening Program, which provides periodic health examinations to eligible National Health Insurance beneficiaries, generally every 2 years and annually for non-office employees. The screening program includes self-administered questionnaires on smoking, alcohol consumption, and physical activity, as well as measurements of height, weight, waist circumference, and blood pressure at each general health examination. Laboratory tests include fasting glucose and serum lipid levels; however, serum lipid testing is performed according to age-, sex-, and screening interval-specific criteria rather than at every examination. Screening institutions submitted the questionnaire and examination results to the KNHIS, where they were de-identified and linked at the individual level with eligibility, claims, diagnosis, procedure, and prescription data. Accordingly, health screening information was available only for individuals who participated in the corresponding examination. The Korean Classification of Diseases, 7th Revision (KCD-7), a modified version of the International Classification of Diseases, 10th Revision (ICD-10), is adapted to the Korean healthcare system. Prescription drug information is classified according to the Korean Standard Classification of Medicines (25).

In this study, three complementary analyses were conducted. First, we evaluated the risk of metabolic diseases among patients with TC who underwent thyroidectomy compared with matched individuals from the general population without a history of TC. Second, among patients with TC, we assessed the risk of metabolic diseases according to thyroidectomy status. Third, among patients with TC who underwent thyroidectomy, we evaluated the risk of metabolic diseases according to thyroid hormone therapy status.

Patients with TC who underwent thyroidectomy were defined as individuals aged 40 to 64 years who received a diagnosis of TC (ICD-10 code: C73) and who underwent either total thyroidectomy or lobectomy between 2007 and 2018. Thyroidectomy was identified through the presence of at least one claim for thyroidectomy-related procedure codes (P4551, P4552, P4553, P4554, or P4561) in the KNHIS claims data (26). Patients in the thyroid hormone user group were defined as those who were prescribed thyroid hormone medication for a total of 1,095 days (three years) or more within the five years following thyroidectomy. Thyroid hormone medications included either T4 monotherapy (levothyroxine sodium) or combination therapy with T4 and T3 (liothyronine sodium) (27).

For the first comparison, individuals without a history of TC were selected from the general population and propensity score–matched (1:4) to patients with TC who underwent thyroidectomy based on age, sex, and income level. The index date for matched individuals was aligned with that of the matched TC patient to ensure comparable follow-up periods.

Participants were excluded if, prior to the index date (defined as the date of thyroidectomy for the surgical cohort and the matched index date for controls), they met any of the following criteria: (1) prior thyroidectomy within the preceding five years; (2) thyroid dysfunction requiring ≥60 days of thyroid hormone therapy; (3) any other malignancy, defined as ≥1 inpatient admission or ≥2 outpatient visits with a cancer diagnosis; (4) chronic kidney disease (N18), heart failure (I50), or liver cirrhosis (K703, K743–K746, K717), each defined as ≥1 inpatient record or ≥2 outpatient records; or (5) prior overweight/obesity, diabetes mellitus, hypertension, dyslipidemia, metabolic syndrome, or MASLD.

### Metabolic abnormalities

Metabolic abnormalities were defined as incident cases identified during the follow-up period among individuals who did not exhibit the respective condition at baseline. These cases were determined through a combination of ICD-10 diagnostic codes derived from medical claims data and corresponding clinical or laboratory measurements obtained from national health screening records. Diagnoses were confirmed if individuals had at least one inpatient admission or two outpatient visits associated with relevant ICD-10 codes, accompanied by objective criteria. Overweight and obesity were classified based on body mass index (BMI), with cutoffs of ≥23 kg/m² and ≥25 kg/m², respectively, in accordance with the Asia-Pacific classification (28). Diabetes mellitus was defined by ICD-10 codes E10–E14 in conjunction with a fasting blood glucose level of ≥126 mg/dL (29). Hypertension was identified through the presence of ICD-10 codes I10–I15, along with systolic blood pressure ≥140 mmHg and diastolic blood pressure ≥90 mmHg (30). Dyslipidemia was diagnosed using ICD-10 code E78 and the presence of at least one abnormal lipid parameter, including total cholesterol ≥240 mg/dL, low-density lipoprotein (LDL) cholesterol ≥160 mg/dL, high-density lipoprotein (HDL) cholesterol <40 mg/dL, or triglycerides ≥200 mg/dL (31, 32). Lipid-lowering medication use was not included in the definition of dyslipidemia. Metabolic syndrome was determined based on modified criteria from the National Cholesterol Education Program Adult Treatment Panel III for Asian populations: individuals with abdominal obesity (waist circumference ≥90 cm in men or ≥80 cm in women) and at least two of the following components—triglycerides ≥150 mg/dL, HDL cholesterol <40 mg/dL in men or <50 mg/dL in women, blood pressure ≥130/85 mmHg, or fasting glucose ≥100 mg/dL—were classified as having metabolic syndrome (33). MASLD was defined using validated surrogate indices: a fatty liver index ≥30 or a hepatic steatosis index >36 (34, 35).

### Covariates

This study considered several confounding variables: age, sex, household income, alcohol consumption, smoking status, and physical activity. Household income served as a proxy for socioeconomic status, determined by the Korean National Health Insurance premium and categorized into quartiles (Q1–Q4), representing the lowest to highest income levels. Alcohol consumption was assessed through self-reported frequency and categorized into five groups: (1) none, (2) ≤1 time per week, (3) ≤2 times per week, (4) ≤4 times per week, and (5) ≥5 times per week. Smoking status was classified into three categories: never smoker, former smoker, and current smoker. Physical activity was measured based on frequency and grouped into five categories: (1) none, (2) ≤2 times per week, (3) ≤4 times per week, (4) ≤6 times per week, and (5) always (daily or near-daily activity).

### Statistical analysis

Descriptive statistics were employed to summarize the characteristics of the study population. Categorical variables are presented as frequencies and percentages, while continuous variables are expressed as medians with interquartile ranges (Q1–Q4). Conditional logistic regression was utilized for matched comparisons. Because metabolic outcomes were identified using both claims and health screening data, the date on which an outcome was first identified according to our operational definition may reflect the timing of clinical or screening-based detection rather than the true disease onset. In addition, actual screening intervals may vary because individuals may undergo screening outside their designated year or skip screening cycles. Given that our objective was to compare the cumulative occurrence of metabolic outcomes within prespecified 5-, 8-, and 10-year follow-up periods, rather than to estimate hazards based on an assumed exact onset date, outcome status at each fixed follow-up period was analyzed using logistic regression. All models were adjusted for age, sex, household income, alcohol consumption, smoking status, and physical activity. Results are presented as odds ratios (ORs) with 95% confidence intervals (CIs), and statistical significance was set at a two-sided p-value of <0.05. Sex-stratified analyses were also conducted to explore potential sex-specific associations. All statistical analyses were performed using R software version 3.6.3 (R Foundation for Statistical Computing, Vienna, Austria).

## Results

### Characteristics of study participants

**Figure 1:**
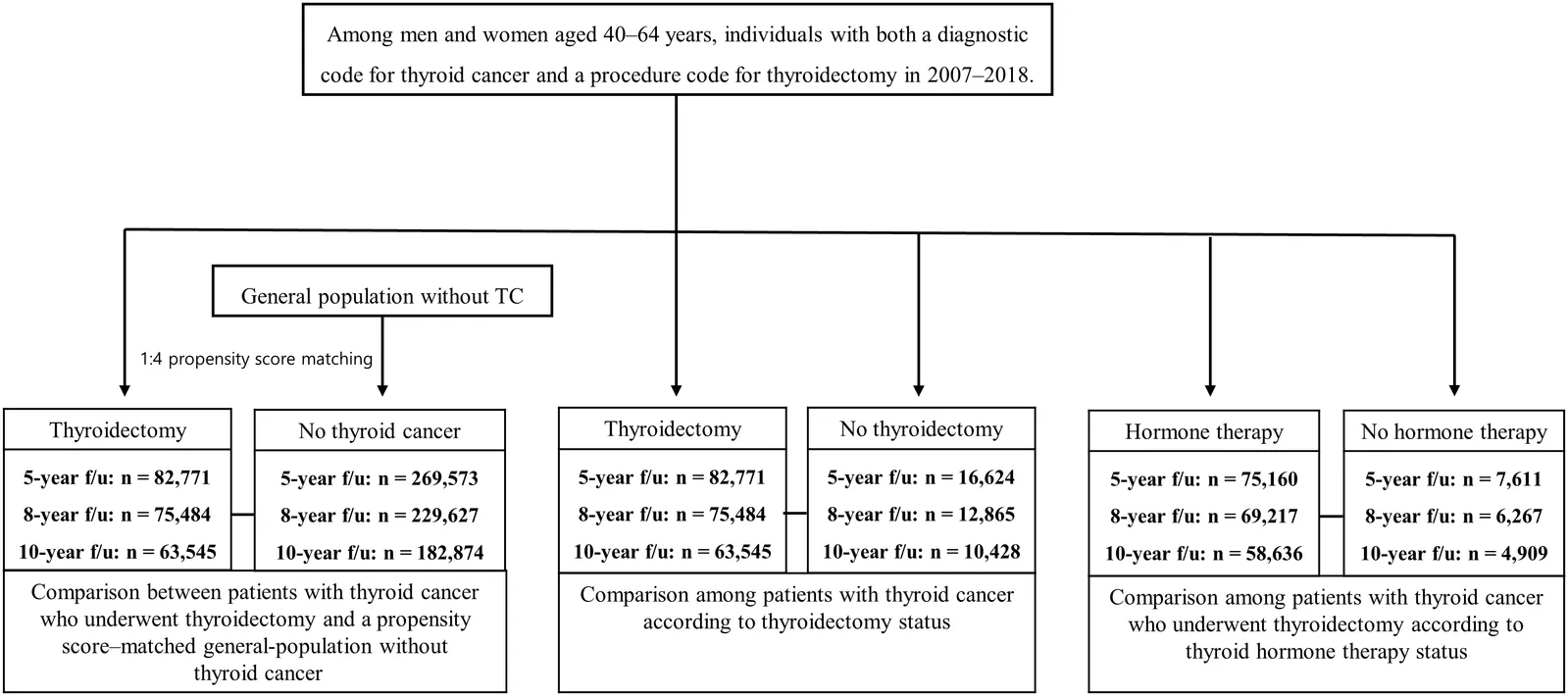
Flowchart of participant inclusion.

The selection process for study participants is illustrated in **Figure 1**. Baseline characteristics and incident metabolic outcomes during follow-up are detailed in **Tables 1**–**3**. **Table 1** compares baseline characteristics and incident metabolic outcomes between patients with TC who underwent thyroidectomy and matched controls from the general population. The total sample sizes were 352,344 for the 5-year follow-up cohort, 305,111 for the 8-year cohort, and 246,419 for the 10-year cohort. **Table 2** compares baseline characteristics and incident metabolic outcomes of patients with TC based on their thyroidectomy status, which included 99,395, 88,349, and 73,973 participants in the 5-, 8-, and 10-year follow-up cohorts, respectively. **Table 3** compares baseline characteristics and incident metabolic outcomes among patients with TC who underwent thyroidectomy, categorized by thyroid hormone therapy use, with 82,771, 75,484, and 63,545 participants in the 5-, 8-, and 10-year follow-up cohorts, respectively.

**Table 1:** Baseline characteristics and incident metabolic outcomes in the thyroidectomy group and a propensity score–matched general-population control group without thyroid cancer.

Characteristic	5 years of follow-up (*n* = 352,344)		8 years of follow-up (*n* = 305,111)		10 years of follow-up (*n* = 246,419)	
	TC with thyroidectomy (*n* = 82,771)	No TC (*n* = 269,573)	TC with thyroidectomy (*n* = 75,484)	No TC (*n* = 229,627)	TC with thyroidectomy (*n* = 63,545)	No TC (*n* = 182,874)
Baseline						
Age, yr ^a^	49.0 (44.0-54.0)	48.0 (43.0-54.0)	49.0 (44.0-54.0)	48.0 (43.0-53.0)	48.0 (43.0-54.0)	48.0 (43.0-53.0)
Sex ^b^						
Men	14,818 (17.9)	29,215 (10.8)	13,258 (17.6)	23,504 (10.2)	11,007 (17.3)	17,563 (9.6)
Women	67,953 (82.1)	240,358 (89.2)	62,226 (82.4)	206,123 (89.8)	52,538 (82.7)	165,311 (90.4)
Household income ^b^						
Q1	1,117 (1.3)	3,925 (1.5)	924 (1.2)	6,408 (1.4)	676 (1.1)	2,446 (1.3)
Q2	28,685 (34.7)	79,910 (29.6)	25,345 (33.6)	67,397 (29.4)	20,785 (32.7)	54,292 (29.7)
Q3	14,880 (18.0)	49,789 (18.5)	13,414 (17.8)	42,481 (18.5)	11,101 (17.5)	33,859 (18.5)
Q4	38,089 (46.0)	135,949 (50.4)	35,801 (47.4)	113,341 (50.7)	30,983 (48.7)	92,277 (50.5)
Alcohol consumption ^b^						
None	53,661 (64.8)	181,238 (67.2)	49,452 (65.5)	156,069 (68.0)	42,070 (66.2)	125,475 (68.6)
≤ once/week	15,439 (18.7)	46,061 (17.1)	14,030 (18.6)	38,635 (16.8)	11,650 (18.3)	30,267 (16.6)
≤ 2 times/week	9,349 (11.3)	25,530 (9.5)	8,327 (11.0)	21,294 (9.3)	6,955 (10.9)	16,714 (9.1)
≤ 4 times/week	3,232 (3.9)	12,086 (4.5)	2,731 (3.6)	9,782 (4.3)	2,113 (3.3)	7,452 (4.1)
≥ 5 times/week	1,090 (1.3)	4,658 (1.7)	944 (1.3)	3,847 (1.7)	757 (1.3)	2,966 (1.6)
Smoking ^b^						
None	69,655 (84.2)	230,840 (85.6)	63,986 (84.8)	198,394 (86.4)	54,216 (85.3)	159,284 (87.1)
Ex-smoker	3,877 (4.7)	10,413 (3.9)	3,392 (4.5)	8,396 (3.7)	2,773 (4.4)	6,428 (3.5)
Current smoker	9,239 (11.1)	28,320 (10.5)	8,106 (10.7)	22,837 (9.9)	6,556 (10.3)	17,162 (9.4)
Physical activity ^b^						
None	52,377 (63.3)	178,990 (66.4)	47,139 (62.4)	151,562 (66.0)	39,330 (61.9)	120,150 (65.7)
≤ 2 times/week	17,510 (21.2)	51,416 (19.1)	16,219 (21.5)	44,024 (19.2)	13,845 (21.8)	35,116 (19.2)
≤ 4 times/week	7,788 (9.3)	23,334 (8.7)	7,339 (9.7)	20,184 (8.8)	6,270 (9.9)	16,351 (8.9)
≤ 6 times/week	2,476 (3.0)	8,153 (3.0)	2,246 (3.0)	7,026 (3.1)	1,882 (3.0)	5,593 (3.1)
Always	2,620 (3.2)	7,680 (2.8)	2,541 (3.4)	6,831 (3.0)	2,218 (3.4)	5,664 (3.1)
BMI (kg/m^2^) ^a^	23.1 (21.2-25.3)	21.3 (20.0-22.7)	23.1 (21.3-25.2)	21.3 (20.0-22.7)	23.1 (21.3-25.2)	21.4 (20.1-22.7)
Systolic BP (mmHg) ^a^	120.0 (110.0-129.0)	115.0 (107.0-125.0)	120.0 (11.00-129.0)	115.0 (107.0-125.0)	120.0 (110.0-129.0)	116.0 (108.0-126.0)
Diastolic BP (mmHg) ^a^	74.0 (70.0-80.0)	70.0 (66.0-80.0)	74.0 (70.0-80.0)	70.0 (66.0-80.0)	74.0 (70.0-80.0)	71.0 (66.0-80.0)
FPG (mg/dL) ^a^	90.0 (83.0-98.0)	90.0 (84.0-98.0)	90.0 (83.0-98.0)	90.0 (84.0-98.0)	90.0 (82.0-98.0)	90.0 (84.0-98.0)
TC (mg/dL) ^a^	189.0 (167.0-212.0)	189.0 (167.0-213.0)	188.0 (167.0-212.0)	190.0 (168.0-214.0)	188.0 (167.0-211.0)	190.0 (168.0-214.0)
AST (U/L) ^a^	21.0 (17.0-25.0)	20.0 (17.0-25.0)	21.0 (17.0-25.0)	20.0 (17.0-25.0)	21.0 (17.0-25.0)	20.0 (17.0-25.0)
ALT (U/L) ^a^	17.0 (13.0-24.0)	17.0 (13.0-22.0)	17.0 (13.0-24.0)	17.0 (13.0-22.0)	17.0 (13.0-24.0)	17.0 (13.0-22.0)
WC (cm) ^a^	77.0 (72.0-83.0)	72.0 (68.0-78.0)	77.0 (72.0-83.0)	72.0 (68.0-78.0)	77.0 (72.0-83.0)	73.0 (68.0-78.0)
TG (mg/dL) ^a^	96.0 (68.0-139.0)	87.0 (63.0-123.0)	96.0 (68.0-139.0)	87.0 (63.0-124.0)	96.0 (68.0-138.0)	88.0 (64.0-12.05)
HDL-C (mg/dL) ^a^	55.0 (47.0-64.0)	59.0 (50.0-69.0)	55.0 (46.0-64.0)	59.0 (50.0-69.0)	55.0 (46.0-64.0)	59.0 (50.0-68.0)
LDL-C (mg/dL) ^a^	115.0 (95.0-137.0)	113.0 (93.0-135.0)	115.0 (95.0-136.0)	113.0 (93.0-136.0)	114.0 (95.0-136.0)	114.0 (94.0-136.0)
Incidence during follow-up						
Overweight/Obesity ^b^						
No	48,029 (58.0)	184,206 (68.3)	38,141 (50.5)	139,362 (60.7)	29,818 (46.9)	103,392 (56.5)
Yes	34,742 (42.0)	85,367 (31.7)	37,343 (49.5)	90,265 (39.3)	33,727 (53.1)	79,482 (43.5)
Diabetes Mellitus ^b^						
No	63,154 (76.3)	261,862 (97.1)	53,170 (70.4)	219,614 (95.6)	42,020 (66.1)	173,082 (94.6)
Yes	19,617 (23.7)	7,711 (2.9)	22,314 (29.6)	10,013 (4.4)	21,525 (33.9)	9,792 (5.4)
Hypertension ^b^						
No	60,841 (73.5)	264,822 (98.2)	51,657 (68.4)	222,352 (96.8)	41,117 (64.7)	175,062 (95.7)
Yes	21,930 (26.5)	4,751 (1.8)	23,827 (31.6)	7,275 (3.2)	22,428 (35.3)	7,812 (4.3)
Dyslipidemia ^b^						
No	75,934 (91.7)	251,119 (93.2)	66,475 (88.1)	204,688 (89.1)	54,805 (86.2)	158,937 (86.9)
Yes	6,837 (8.3)	18,454 (6.8)	9,009 (11.9)	24,939 (10.9)	8,740 (13.8)	23,937 (13.1)
Metabolic Syndrome ^b^						
No	72,430 (87.5)	247,626 (91.9)	62,910 (83.3)	201,466 (87.7)	51,709 (81.4)	156,063 (85.3)
Yes	10,341 (12.5)	21,947 (8.1)	12,574 (16.7)	28,161 (12.3)	11,836 (18.6)	26,811 (14.7)
MASLD ^b^						
No	57,155 (69.1)	233,319 (86.6)	52,093 (69.0)	198,165 (86.3)	43,768 (68.9)	156,820 (85.8)
Yes	25,616 (30.9)	36,254 (13.4)	23,391 (31.0)	31,462 (13.7)	19,777 (31.1)	26,054 (14.2)

TC, Thyroid cancer; MASLD, metabolic dysfunction-associated steatotic liver disease; BMI, body mass index; SBP, Systolic BP; DBP, Diastolic BP; FPG, fasting plasma glucose; TC, total cholesterol; AST, aspartate aminotransferase; ALT, alanine aminotransferase; WC, waist circumference; TG, triglyceride; HDL-C, high-density lipoprotein cholesterol; LDL-C, low-density lipoprotein cholesterol.

^a^Data are presented as median (Q1–Q4).

^b^Data are presented as N(%).

**Table 2:** Baseline characteristics and incident metabolic outcomes in thyroid cancer patients with and without thyroidectomy.

Characteristic	5 years of follow-up (*n* = 99,395)		8 years of follow-up (*n* = 88,349)		10 years of follow-up (*n* = 73,973)	
	Thyroidectomy (*n* = 82,771)	No thyroidectomy (*n* = 16,624)	Thyroidectomy (*n* = 75,484)	No thyroidectomy (*n* = 12,865)	Thyroidectomy (*n* = 63,545)	No thyroidectomy (*n* = 10,428)
Baseline						
Age, yr ^a^	49.0 (44.0-54.0)	48.0 (43.0-54.0)	49.0 (44.0-54.0)	48.0 (43.0-54.0)	48.0 (43.0-54.0)	48.0 (43.0-54.0)
Sex ^b^						
Men	14,818 (17.9)	3,043 (18.3)	13,258 (17.6)	2,279 (17.7)	11,007 (17.3)	1,809 (17.3)
Women	67,953 (82.1)	13,581 (81.7)	62,226 (82.4)	10,586 (82.3)	52,538 (82.7)	8,619 (82.7)
Household income ^b^						
Q1	1,117 (1.3)	302 (1.8)	924 (1.2)	209 (1.6)	676 (1.1)	162 (1.6)
Q2	28,685 (34.7)	5,411 (32.5)	25,345 (33.6)	4,003 (31.1)	20,785 (32.7)	3,124 (30.0)
Q3	14,880 (18.0)	2,823 (17.0)	13,414 (17.8)	2,133 (16.6)	11,101 (17.5)	1,714 (16.4)
Q4	38,089 (46.0)	8,088 (48.7)	35,801 (47.4)	6,520 (50.7)	30,983 (48.7)	5,428 (52.0)
Alcohol consumption ^b^						
None	53,661 (64.8)	10,760 (64.7)	49,452 (65.5)	8,421 (65.5)	42,070 (66.2)	6,856 (65.7)
≤ once/week	15,439 (18.7)	3,166 (19.0)	14,030 (18.6)	2398 (18.6)	11,650 (18.3)	1,913 (18.3)
≤ 2 times/week	9,349 (11.3)	1,793 (10.8)	8,327 (11.0)	1,377 (10.7)	6,955 (10.9)	1,107 (10.6)
≤ 4 times/week	3,232 (3.9)	671 (4.1)	2,731 (3.6)	501 (3.9)	2,113 (3.3)	414 (4.1)
≥ 5 times/week	1,090 (1.3)	234 (1.4)	944 (1.3)	168 (1.3)	757 (1.3)	138 (1.3)
Smoking ^b^						
None	69,655 (84.2)	13,859 (83.4)	63,986 (84.8)	10,838 (84.2)	54,216 (85.3)	8,846 (84.8)
Ex-smoker	3,877 (4.7)	874 (5.3)	3,392 (4.5)	657 (5.1)	2,773 (4.4)	501 (4.8)
Current smoker	9,239 (11.1)	1,891 (11.3)	8,106 (10.7)	1,370 (10.7)	6,556 (10.3)	1,081 (10.4)
Physical activity ^b^						
None	52,377 (63.3)	10,543 (63.4)	47,139 (62.4)	8,013 (62.3)	39,330 (61.9)	6,469 (62.0)
≤ 2 times/week	17,510 (21.2)	3,532 (21.2)	16,219 (21.5)	2,801 (21.8)	13,845 (21.8)	2,273 (21.8)
≤ 4 times/week	7,788 (9.3)	1,630 (9.8)	7,339 (9.7)	1,304 (10.1)	6,270 (9.9)	1,068 (10.2)
≤ 6 times/week	2,476 (3.0)	458 (2.8)	2,246 (3.0)	368 (2.9)	1,882 (3.0)	295 (2.8)
Always	2,620 (3.2)	461 (2.8)	2,541 (3.4)	379 (2.9)	2,218 (3.4)	323 (3.2)
BMI (kg/m^2^) ^a^	23.1 (21.2-25.3)	22.8 (21.0-25.0)	23.1 (21.3-25.2)	22.9 (21.1-25.0)	23.1 (21.3-25.2)	22.9 (21.1-25.0)
Systolic BP (mmHg) ^a^	120.0 (110.0-129.0)	119.0 (110.0-129.0)	120.0 (110.0-129.0)	119.0 (110.0-129.0)	120.0 (110.0-129.0)	119.0 (110.0-129.0)
Diastolic BP (mmHg) ^a^	74.0 (70.0-80.0)	73.0 (69.0-80.0)	74.0 (70.0-80.0)	73.0 (69.0-80.0)	74.0 (70.0-80.0)	73.0 (69.0-80.0)
FPG (mg/dL) ^a^	90.0 (83.0-98.0)	90.0 (83.0-98.0)	90.0 (83.0-98.0)	90.0 (82.0-98.0)	90.0 (82.0-98.0)	90.0 (82.0-98.0)
TC (mg/dL) ^a^	189.0 (167.0-212.0)	189.0 (167.0-213.0)	188.0 (167.0-212.0)	189.0 (167.0-213.0)	188.0 (167.0-211.0)	189.0 (167.0-213.0)
AST (U/L) ^a^	21.0 (17.0-25.0)	20.0 (17.0-25.0)	21.0 (17.0-25.0)	20.0 (17.0-25.0)	21.0 (17.0-25.0)	20.0 (17.0-25.0)
ALT (U/L) ^a^	17.0 (13.0-24.0)	17.0 (13.0-24.0)	17.0 (13.0-24.0)	17.0 (13.0-24.0)	17.0 (13.0-24.0)	17.0 (13.0-24.0)
WC (cm) ^a^	77.0 (72.0-83.0)	76.0 (71.0-83.0)	77.0 (72.0-83.0)	76.0 (71.0-83.0)	77.0 (72.0-83.0)	76.0 (71.0-83.0)
TG (mg/dL) ^a^	96.0 (68.0-139.0)	95.0 (67.0-138.0)	96.0 (68.0-139.0)	95.0 (67.0-136.0)	96.0 (68.0-138.0)	95.0 (67.0-136.0)
HDL-C (mg/dL) ^a^	55.0 (47.0-64.0)	55.0 (47.0-65.0)	55.0 (46.0-64.0)	55.0 (47.0-65.0)	55.0 (46.0-64.0)	55.0 (47.0-64.0)
LDL-C (mg/dL) ^a^	115.0 (95.0-137.0)	115.0 (95.0-137.0)	115.0 (95.0-136.0)	115.0 (96.0-137.0)	114.0 (95.0-136.0)	115.0 (96.0-137.0)
Incidence during follow-up						
Overweight/Obesity ^b^						
No	48,029 (58.0)	9,032 (54.3)	38,141 (50.5)	6,261 (48.7)	29,818 (46.9)	4,820 (46.2)
Yes	34,742 (42.0)	7,592 (45.7)	37,343 (49.5)	6,604 (51.3)	33,727 (53.1)	5,608 (53.8)
Diabetes Mellitus ^b^						
No	63,154 (76.3)	14,673 (88.3)	53,170 (70.4)	10,629 (82.6)	42,020 (66.1)	8,356 (80.1)
Yes	19,617 (23.7)	1,951 (11.7)	22,314 (29.6)	2,236 (17.4)	21,525 (33.9)	2,072 (19.9)
Hypertension ^b^						
No	60,841 (73.5)	14,229 (85.6)	51,657 (68.4)	10,252 (79.7)	41,117 (64.7)	8,034 (77.0)
Yes	21,930 (26.5)	2,395 (14.4)	23,827 (31.6)	2,613 (20.3)	22,428 (35.3)	2,394 (23.0)
Dyslipidemia ^b^						
No	75,934 (91.7)	15,236 (91.7)	66,475 (88.1)	11,327 (88.0)	54,805 (86.2)	9,009 (86.4)
Yes	6,837 (8.3)	1,388 (8.3)	9,009 (11.9)	1,538 (12.0)	8,740 (13.8)	1,419 (13.6)
Metabolic Syndrome ^b^						
No	72,430 (87.5)	14,553 (87.5)	62,910 (83.3)	10,770 (83.7)	51,709 (81.4)	8,524 (81.7)
Yes	10,341 (12.5)	2,071 (12.5)	12,574 (16.7)	2,095 (16.3)	11,836 (18.6)	1,904 (18.3)
MASLD ^b^						
No	57,155 (69.1)	11,983 (72.1)	52,093 (69.0)	9,270 (72.1)	43,768 (68.9)	7,510 (72.0)
Yes	25,616 (30.9)	4,641 (27.9)	23,391 (31.0)	3,595 (27.9)	19,777 (31.1)	2,918 (28.0)

TC, Thyroid cancer; MASLD, metabolic dysfunction-associated steatotic liver disease; BMI, body mass index; SBP, Systolic BP; DBP, Diastolic BP; FPG, fasting plasma glucose; TC, total cholesterol; AST, aspartate aminotransferase; ALT, alanine aminotransferase; WC, waist circumference; TG, triglyceride; HDL-C, high-density lipoprotein cholesterol; LDL-C, low-density lipoprotein cholesterol.

^a^Data are presented as median (Q1–Q4).

^b^Data are presented as N(%).

**Table 3:** Baseline characteristics and incident metabolic outcomes in thyroid cancer patients with thyroidectomy by thyroid hormone therapy use.

Characteristic	5 years of follow-up (*n* = 82,771)		8 years of follow-up (*n* = 75,484)		10 years of follow-up (*n* = 63,545)	
	Thyroid hormone therapy (*n* = 75,160)	No thyroid hormone therapy (*n* = 7,611)	Thyroid hormone therapy (*n* = 69,217)	No thyroid hormone therapy (*n* = 6,267)	Thyroid hormone therapy (*n* = 58,636)	No thyroid hormone therapy (*n* = 4,909)
Baseline						
Age, yr ^a^	49.0 (44.0-54.0)	48.0 (43.0-53.0)	49.0 (44.0-54.0)	48.0 (43.0-53.0)	49.0 (43.0-54.0)	48.0 (43.0-52.0)
Sex ^b^						
Men	13,367 (17.8)	1,451 (19.1)	12,066 (17.4)	1,192 (19.0)	10,090 (17.2)	917 (18.7)
Women	61,793 (82.2)	6,160 (80.9)	57,151 (82.6)	5,075 (81.0)	48,546 (82.8)	3,992 (81.3)
Household income ^b^						
Q1	1,009 (1.3)	108 (1.4)	842 (1.2)	82 (1.3)	615 (1.0)	61 (1.2)
Q2	26,029 (34.6)	2,656 (34.9)	23,265 (33.6)	2,080 (33.2)	19,176 (32.7)	1,609 (32.8)
Q3	13,524 (18.0)	1,356 (17.8)	12,310 (17.8)	1,104 (17.6)	10,235 (17.5)	866 (17.6)
Q4	34,598 (46.1)	3,491 (45.9)	32,800 (47.4)	3,001 (47.9)	28,610 (48.8)	2,373 (48.3)
Alcohol consumption ^b^						
None	48,837 (65.0)	4,824 (63.4)	45,384 (65.6)	4,068 (64.9)	38,833 (66.2)	3,237 (65.9)
≤ once/week	13,971 (18.6)	1,468 (19.3)	12,856 (18.6)	1,174 (18.8)	10,762 (18.4)	888 (18.1)
≤ 2 times/week	8,467 (11.3)	882 (11.6)	7,633 (11.0)	694 (11.1)	6,403 (10.9)	552 (11.2)
≤ 4 times/week	2,895 (3.9)	337 (4.4)	2,472 (3.6)	259 (4.1)	1,929 (3.3)	184 (3.7)
≥ 5 times/week	990 (1.2)	100 (1.3)	872 (1.2)	72 (1.1)	709 (1.2)	48 (1.0)
Smoking ^b^						
None	63,336 (84.3)	6,319 (83.0)	58,747 (84.9)	5,239 (83.6)	50,074 (85.4)	4,142 (84.4)
Ex-smoker	3,498 (4.7)	379 (5.0)	3,096 (4.4)	296 (4.7)	2,548 (4.3)	225 (4.6)
Current smoker	8,326 (11.0)	913 (12.0)	7,374 (10.7)	732 (11.7)	6,014 (10.3)	542 (11.0)
Physical activity ^b^						
None	47,512 (63.2)	4,865 (63.9)	43,212 (62.4)	3,927 (62.7)	36,294 (61.9)	3,036 (61.8)
≤ 2 times/week	15,921 (21.2)	1,589 (20.9)	14,895 (21.5)	1,324 (21.1)	12,778 (21.8)	1,067 (21.7)
≤ 4 times/week	7,049 (9.4)	739 (9.7)	6,695 (9.7)	644 (10.3)	5,754 (9.8)	516 (10.6)
≤ 6 times/week	2,275 (3.0)	201 (2.6)	2,066 (3.0)	180 (2.9)	1,745 (3.0)	137 (2.8)
Always	2,403 (3.2)	217 (2.9)	2,349 (3.4)	192 (3.0)	2,065 (3.5)	153 (3.1)
BMI (kg/m^2^) ^a^	23.1 (21.3-25.3)	22.8 (21.0-25.0)	23.1 (21.3-25.2)	22.8 (21.1-25.0)	23.1 (21.3-25.2)	22.8 (21.1-24.9)
Systolic BP (mmHg) ^a^	120.0 (110.0-130.0)	118.0 (110.0-127.0)	120.0 (110.0-129.0)	118.0 (110.0-127.0)	120.0 (110.0-129.0)	118.0 (110.0-127.0)
Diastolic BP (mmHg) ^a^	74.0 (70.0-80.0)	73.0 (69.0-80.0)	74.0 (70.0-80.0)	72.0 (69.0-80.0)	74.0 (70.0-80.0)	72.0 (69.0-80.0)
FPG (mg/dL) ^a^	90.0 (83.0-98.0)	90.0 (83.0-98.0)	90.0 (82.0-98.0)	90.0 (83.0-98.0)	90.0 (82.0-98.0)	90.0 (83.0-98.0)
TC (mg/dL) ^a^	189.0 (167.0-212.0)	188.0 (167.0-212.0)	188.0 (167.0-212.0)	188.0 (167.0-212.0)	188.0 (167.0-211.0)	188.0 (167.0-211.0)
AST (U/L) ^a^	21.0 (17.0-25.0)	20.0 (17.0-25.0)	21.0 (17.0-25.0)	20.0 (17.0-25.0)	21.0 (17.0-25.0)	20.0 (17.0-25.0)
ALT (U/L) ^a^	17.0 (13.0-24.0)	17.0 (13.0-24.0)	17.0 (13.0-24.0)	17.0 (13.0-24.0)	17.0 (13.0-24.0)	17.0 (13.0-24.0)
WC (cm) ^a^	77.0 (72.0-83.0)	76.0 (71.0-83.0)	77.0 (72.0-83.0)	76.0 (71.0-83.0)	77.0 (72.0-83.0)	76.0 (71.0-83.0)
TG (mg/dL) ^a^	96.0 (68.0-139.0)	96.0 (67.0-140.0)	96.0 (68.0-138.0)	96.0 (67.0-139.0)	96.0 (68.0-138.0)	96.0 (67.0-139.0)
HDL-C (mg/dL) ^a^	55.0 (47.0-64.0)	55.0 (47.0-64.0)	55.0 (47.0-64.0)	55.0 (47.0-64.0)	55.0 (46.0-64.0)	55.0 (47.0-64.0)
LDL-C (mg/dL) ^a^	114.0 (95.0-137.0)	114.0 (95.0-137.0)	115.0 (95.0-136.0)	114.0 (95.0-137.0)	114.0 (95.0-136.0)	115.0 (96.0-137.0)
Incidence during follow-up						
Overweight/Obesity ^b^						
No	43,376 (57.7)	4,653 (61.1)	34,733 (50.2)	3,408 (54.4)	27,292 (46.5)	2,526 (51.5)
Yes	31,784 (42.3)	2,958 (38.9)	34,484 (49.8)	2,859 (45.6)	31,344 (53.5)	2,383 (48.5)
Diabetes Mellitus ^b^						
No	55,824 (74.3)	7,330 (96.3)	47,240 (68.2)	5,930 (94.6)	37,433 (63.8)	4,587 (93.4)
Yes	19,336 (25.7)	281 (3.7)	21,977 (31.8)	337 (5.4)	21,203 (36.2)	322 (6.6)
Hypertension ^b^						
No	53,478 (71.2)	7,363 (96.7)	45,694 (66.0)	5,963 (95.1)	36,479 (62.2)	4,638 (94.5)
Yes	21,682 (28.8)	248 (3.3)	23,523 (34.0)	304 (4.9)	22,157 (37.8)	271 (5.5)
Dyslipidemia ^b^						
No	68,914 (91.7)	7,020 (92.2)	60,899 (88.0)	5,576 (89.0)	50,544 (86.2)	4,261 (86.8)
Yes	6,246 (8.3)	591 (7.8)	8,318 (12.0)	691 (11.0)	8,092 (13.8)	648 (13.2)
Metabolic Syndrome ^b^						
No	65,647 (87.3)	6,783 (89.1)	57,572 (83.2)	5,338 (85.2)	47,604 (81.2)	4,105 (83.6)
Yes	9,513 (12.7)	828 (10.9)	11,645 (16.8)	929 (14.8)	11,032 (18.8)	804 (16.4)
MASLD ^b^						
No	51,682 (70.8)	5,473 (71.9)	47,574 (68.7)	4,519 (72.1)	40,208 (68.6)	3,560 (72.5)
Yes	23,478 (29.2)	2,138 (28.1)	21,643 (31.3)	1,748 (27.9)	18,428 (31.4)	1,349 (27.5)

MASLD, metabolic dysfunction-associated steatotic liver disease; BMI, body mass index; SBP, Systolic BP; DBP, Diastolic BP; FPG, fasting plasma glucose; TC, total cholesterol; AST, aspartate aminotransferase; ALT, alanine aminotransferase; WC, waist circumference; TG, triglyceride; HDL-C, high-density lipoprotein cholesterol; LDL-C, low-density lipoprotein cholesterol.

^a^Data are presented as median (Q1–Q4).

^b^Data are presented as N(%).

Baseline characteristics were comparable across groups in all three comparisons (**Tables 1**–**3**). During follow-up, compared to matched general-population controls (**Table 1**), the thyroidectomy group exhibited a higher incidence of all metabolic outcomes. Within the TC cohort (**Table 2**), thyroidectomy was associated with an increased incidence of diabetes, hypertension, and MASLD, while the prevalence of overweight/obesity was slightly lower, and dyslipidemia and metabolic syndrome showed similar rates. Among patients who underwent thyroidectomy (**Table 3**), thyroid hormone therapy was linked to a higher incidence of all metabolic outcomes.

### Logistic regression

**Figure 2:**
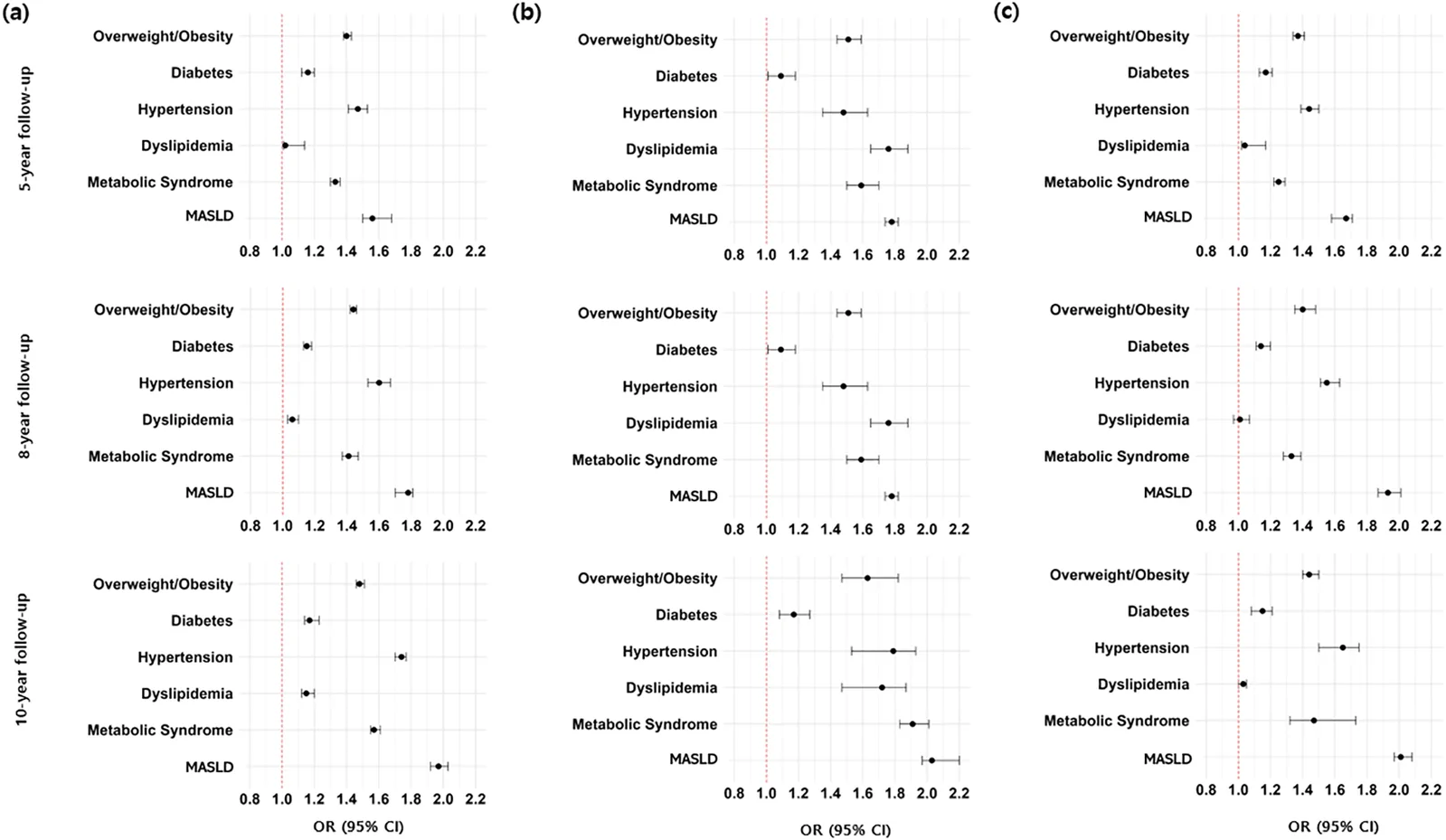
Adjusted odds ratios for metabolic diseases in the thyroidectomy group compared with a propensity score–matched general-population control group without thyroid cancer. Models were adjusted for age, sex, household income, alcohol consumption, smoking status, and physical activity. **(a)** Total population; **(b)** Male participants; **(c)** Female participants. The number of events for each outcome at each follow-up period is provided in **Table 1**. MASLD, metabolic dysfunction-associated steatotic liver disease; OR, odds ratio; CI, confidence interval.

**Figure 2** presents adjusted OR from conditional logistic regression comparing patients with TC who underwent thyroidectomy with a propensity score–matched control group from the general population. In the overall sample (**Figure 2a**), thyroidectomy was associated with higher odds of overweight/obesity, diabetes, hypertension, metabolic syndrome, and MASLD at the 5-, 8-, and 10-year follow-ups (all p<0.001). Dyslipidemia was not significant at 5 years (OR 1.01, 95% CI 0.98–1.04; p=0.494) but became significant at the 8- and 10-year follow-ups. When stratified by sex (**Figures 2b, c**), the overall patterns were consistent for both males and females; however, dyslipidemia was significant in males across all follow-up periods, while in females it was significant at the 5- and 10-year follow-ups but not at 8 years (OR: 1.01, 95% CI: 0.97–1.07, p = 0.711).

**Figure 3:**
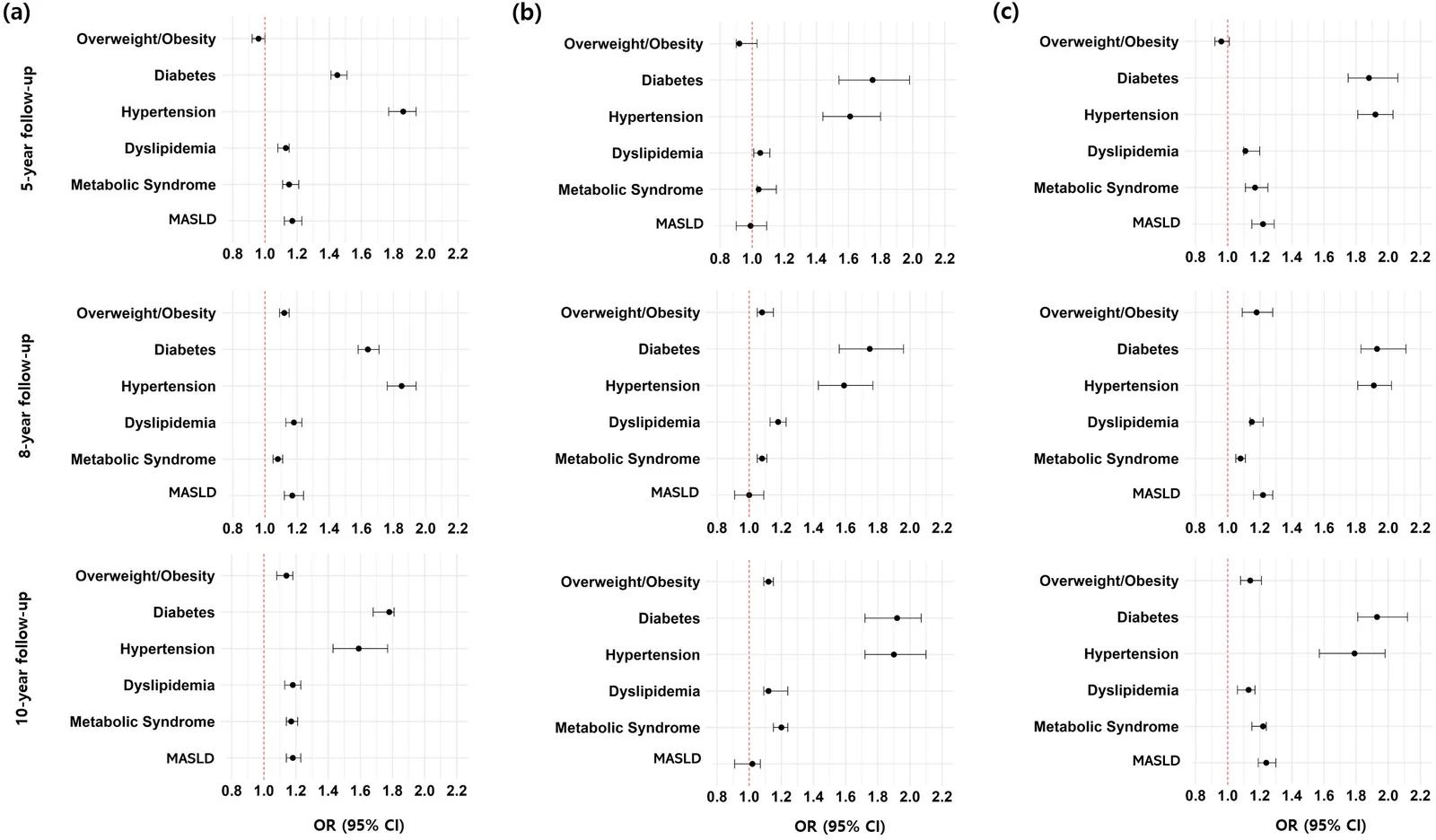
Adjusted odds ratios for metabolic diseases in patients with thyroid cancer who underwent thyroidectomy compared with those who did not. Models were adjusted for age, sex, household income, alcohol consumption, smoking status, and physical activity. **(a)** Total population; **(b)** Male participants; **(c)** Female participants. The number of events for each outcome at each follow-up period is provided in **Table 2**. MASLD, metabolic dysfunction-associated steatotic liver disease; OR, odds ratio; CI, confidence interval.

**Figure 3** displays adjusted OR from logistic regression comparing patients with TC who underwent thyroidectomy to those who did not, within the TC cohort. In the overall sample (**Figure 3a**), thyroidectomy was associated with higher odds of most metabolic diseases across the 5-, 8-, and 10-year follow-ups. The association with overweight/obesity was not statistically significant at 5 years (OR 0.96, 95% CI 0.92–1.00; p=0.092) but reached significance at 8 and 10 years. In sex-stratified analyses (**Figures 3b, c**), the patterns were generally similar; however, MASLD was not significant in males at any follow-up, while significant, persistent associations were observed in females across all time points.

**Figure 4:**
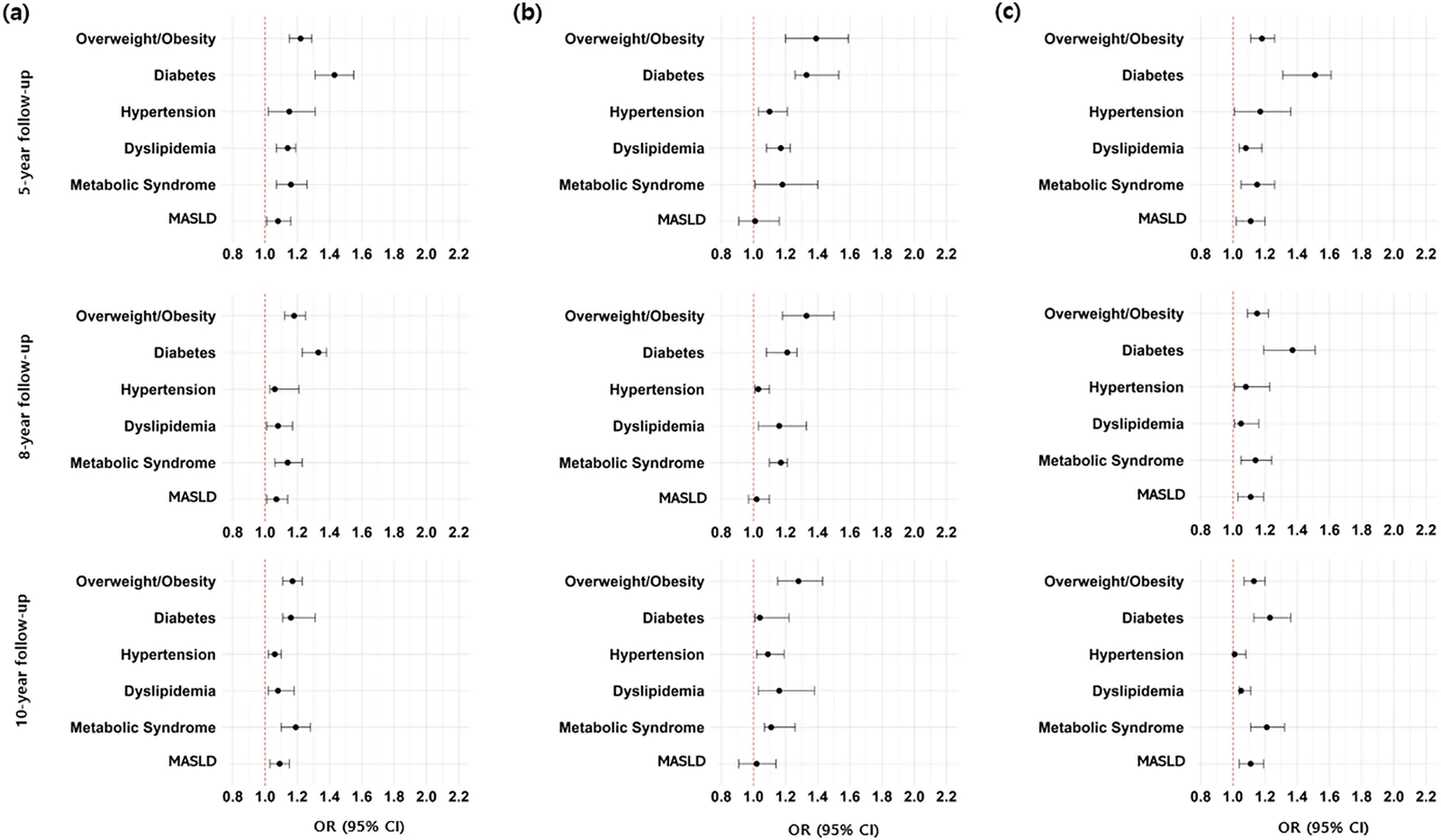
Adjusted odds ratios for metabolic diseases in patients with thyroid cancer who underwent thyroidectomy and received thyroid hormone therapy compared with those not receiving therapy. Models were adjusted for age, sex, household income, alcohol consumption, smoking status, and physical activity. **(a)** Total population; **(b)** Male participants; **(c)** Female participants. The number of events for each outcome at each follow-up period is provided in **Table 3**. MASLD, metabolic dysfunction-associated steatotic liver disease; OR, odds ratio; CI, confidence interval.

**Figure 4** presents adjusted OR derived from logistic regression analyses, comparing patients with TC who underwent thyroidectomy and received thyroid hormone therapy to those who did not receive such therapy. In the overall sample (**Figure 4a**), the administration of thyroid hormone therapy was associated with all metabolic abnormalities at 5, 8, and 10 years post-surgery. The sex-stratified results (**Figures 4b, c**) demonstrated generally consistent findings; however, notable sex-specific differences were identified for MASLD. Specifically, MASLD was not significant in males at any time point, while it remained significant and persistent in females across all follow-up periods.

## Discussion

In our nationwide retrospective cohort study utilizing the Korea National Health Insurance Service (KNHIS) database, we found that thyroidectomy and subsequent thyroid hormone therapy were significantly associated with an incidence of various metabolic disorders, including diabetes mellitus, hypertension, dyslipidemia, metabolic syndrome, and MASLD. These associations persisted over 5-, 8-, and 10-year follow-up periods, even after adjusting for factors such as age, sex, socioeconomic status, and lifestyle. To progressively isolate the contribution of each factor to this metabolic risk, our study employed three distinct comparisons: the comparison with the general population captures the overall impact of having TC and undergoing thyroidectomy; the comparison within the TC cohort by thyroidectomy status evaluates the effect of surgery itself while holding TC diagnosis constant; and the comparison within the thyroidectomy cohort by hormone therapy status evaluates the additional contribution of long-term hormone therapy while holding surgical status constant.

Our findings corroborate earlier reports indicating a relationship between thyroid dysfunction and metabolic disorders (10–14), illustrating that this risk persists even after hormone replacement following thyroidectomy. Previous studies have primarily focused on spontaneous hypo- or hyperthyroidism, often with limited sample sizes or follow-up durations (15–20). In contrast, our study included over 350,000 individuals with standardized national health screening data, allowing for robust estimations of long-term metabolic risks. The consistent increase in the incidence of diabetes, hypertension, and MASLD among patients who underwent thyroidectomy—when compared to the general population as well as patients with TC who did not undergo thyroidectomy—is consistent with the possibility that surgical loss of thyroid function and subsequent hormonal changes may contribute to the observed associations. The observed association between long-term thyroid hormone therapy and metabolic disturbance should be interpreted with caution, as thyroid hormone therapy may represent a marker of underlying disease characteristics rather than an independent exposure. Patients receiving long-term thyroid hormone therapy are likely to differ from those not receiving therapy with respect to factors such as the extent of thyroidectomy, recurrence risk, radioactive iodine treatment, and TSH suppression strategy, all of which may have contributed to the observed association. Nevertheless, several biological mechanisms may also be consistent with the observed association, although these hypotheses could not be directly evaluated in the present study. Excessive thyroid hormone exposure or prolonged TSH suppression, as well as differences between exogenous hormone replacement after thyroidectomy and endogenous thyroid hormone physiology may be consistent with the observed association (36, 37). Consistent with these hypotheses, deviations from thyroid hormone homeostasis have been associated with systemic metabolic effects (7, 8, 17, 38). Subclinical hyperthyroidism resulting from TSH-suppressive therapy can enhance hepatic gluconeogenesis, increase lipolysis, elevate blood pressure, and impair insulin sensitivity (7, 39). Conversely, transient hypothyroidism during radioiodine preparation or levothyroxine dose adjustments, as well as the proposed but not yet firmly established concept of tissue-specific hypothyroidism despite laboratory-confirmed euthyroidism, may contribute to dyslipidemia, weight gain, and MASLD (40). Beyond physiological factors, behavioral changes following a cancer diagnosis—such as decreased physical activity, psychological stress, and altered dietary habits—may synergistically increase metabolic risk (41).

Notably, our sex-stratified analyses revealed a significant association between thyroidectomy and the incidence of hepatic steatosis specifically in female patients. Several mechanisms may contribute to this sex-specific vulnerability. Firstly, thyroidectomy is associated with a reduction in endogenous T3 levels, and this relative deficiency may have a disproportionately greater metabolic impact on women, who appear more susceptible to metabolic disturbances associated with hypothyroidism than men (42, 43). Hepatic lipid metabolism is influenced by both thyroid hormone signaling and estrogen-mediated pathways. The disruption of this estrogen-thyroid-liver axis, particularly among women aged 40 to 60 years who experience declining estrogen levels, may exacerbate steatogenic processes (44, 45). These hormonal changes may partly explain the observed association between thyroidectomy and hepatic fat accumulation in women.

Interestingly, the patterns of dyslipidemia also varied by sex. While women exhibited a trend toward increased dyslipidemia after thyroidectomy, men showed a more pronounced risk. This discrepancy may reflect intrinsic differences in lipid metabolism between the sexes. Estrogen promotes favorable lipid profiles by enhancing hepatic LDL receptor expression and modulating VLDL metabolism (46), which may mitigate the dyslipidemic changes associated with thyroidectomy in women. Conversely, lipid metabolism in men is more significantly influenced by visceral adiposity (47, 48), suggesting that alterations in thyroid hormone dynamics following thyroidectomy may be more closely associated with serum lipid levels in men.

The findings of this study carry significant clinical implications. Clinicians should be aware that patients with thyroid cancer—particularly those undergoing thyroidectomy and long-term hormone therapy—may represent an increased risk for metabolic complications. Routine monitoring for obesity, diabetes, dyslipidemia, and MASLD may warrant consideration in follow-up care. Additionally, given the observational nature of our findings, any change to thyroid hormone dosing strategy would be premature without further studies specifically designed to evaluate whether metabolic risk varies according to the intensity or duration of thyroid hormone therapy; our findings instead support increased attention to metabolic monitoring in patients receiving long-term therapy, rather than an immediate change in prescribing practice (36). As noted above, this tissue-level hyposensitivity remains an evolving hypothesis (36, 49); if confirmed, reliable biomarkers capable of detecting such hyposensitivity could support more personalized treatment strategies and help avert adverse metabolic outcomes. Finally, interdisciplinary management involving endocrinologists, oncologists, and primary care physicians may be beneficial. Lifestyle interventions including weight management, regular physical activity, and healthy dietary practices should be considered to support metabolic health (41).

Several limitations of this study warrant acknowledgment. First, as an observational study, it cannot establish causality, and residual confounding from unmeasured factors—such as dietary habits, family history of metabolic disease, menopausal status, and concurrent medications—cannot be excluded. Second, confounding by indication may influence the results regarding thyroidectomy status and thyroid hormone therapy. Patients undergoing surgery or receiving long-term, intensive therapy likely had more advanced baseline disease or higher recurrence risk. In contrast, the non-thyroidectomy group may have included patients managed with active surveillance, those treated with radiofrequency ablation, and patients with clinically presumed thyroid malignancy who did not undergo surgery, some of whom were likely to have low-risk papillary thyroid microcarcinomas. Because our database lacked granular clinical details (e.g., tumor stage, exact extent of surgery, radioactive iodine treatment, and specific TSH suppression targets), we could not fully differentiate the metabolic impacts of the interventions from those of the underlying disease severity. Furthermore, due to the lack of serum thyroid function tests, levothyroxine doses, and adherence data, thyroid hormone therapy was analyzed strictly as a binary exposure. This prevented a dose-response analysis and restricted our ability to distinguish whether the observed metabolic risks stem from overtreatment, undertreatment, or thyroid hormone use itself. Third, the potential for surveillance bias exists, as thyroid cancer survivors undergo more frequent clinical follow-up and may have higher health screening compliance. We partially mitigated this by utilizing objective biometric criteria from Korea’s universal, biennial national health screening program rather than relying solely on claims-based diagnostic codes; however, differential healthcare-seeking behavior cannot be entirely ruled out. Fourth, our findings may lack generalizability to Western populations, which differ significantly from the Korean population regarding obesity prevalence, iodine intake, thyroid hormone prescribing practices, and the high proportion of small, screening-detected tumors in Korea. Finally, because metabolic outcomes were identified through claims and health screenings, the documented onset may reflect detection timing rather than true physiological onset. While we addressed this by evaluating outcomes within fixed 5-, 8-, and 10-year intervals, individual variations in exact onset timing could not be fully captured. Additionally, MASLD was defined using validated surrogate indices and Asian-specific BMI and waist circumference cutoffs, which limits direct absolute rate comparisons with Western cohorts, though relative associations remain informative.

Despite these limitations, the sheer scale of this cohort—comprising over 350,000 individuals with 10 years of follow-up—provides a level of robust longitudinal evidence that remains scarce in the current literature. The use of objective national screening data and a consistent analytical design further lend significant weight to the reliability of our conclusions.

In conclusion, thyroidectomy and long-term thyroid hormone therapy were associated with subsequent metabolic abnormalities among patients with thyroid cancer. These findings suggest that metabolic health may warrant consideration in the long-term management of this population, including closer metabolic monitoring while future studies evaluate individualized hormone therapy strategies. Although causal inference is limited by the observational design, the large-scale longitudinal evidence from this study provides a valuable basis for improving metabolic risk assessment. Future research should further evaluate the roles of thyroid hormone levels, treatment intensity, and metabolic biomarkers to support more precise and individualized management strategies.

## Data Availability

The original contributions presented in the study are included in the article/supplementary material. Further inquiries can be directed to the corresponding author.
